# 2-Chloro-*N*-{3-cyano-1-[2,6-dichloro-4-(trifluoro­meth­yl)phen­yl]-1*H*-pyrazol-5-yl}acetamide

**DOI:** 10.1107/S1600536811052743

**Published:** 2011-12-14

**Authors:** Jian-qiang Zhang, Qiu He, Qiang-hua Jiang, Hai-pin Mu, Rong Wan

**Affiliations:** a Department of Applied Chemistry, College of Science, Nanjing University of Technology, No. 5 Xinmofan Road, Nanjing, Nanjing 210009, People’s Republic of China

## Abstract

The title compound, C_13_H_6_Cl_3_F_3_N_4_O, was synthesized by the reaction of 5-amino-1-[2,6-dichloro-4-(trifluoro­meth­yl)phen­yl]-1*H*-pyrazole-3-carbonitrile and 2-chloro­acetyl chloride. The five-membered pyrazole ring makes a dihedral angle of 71.5 (3)° with the benzene ring. The –CF_3_ group is disordered by rotation, and the F atoms are split over two sets of sites with occupancies of 0.59 (2) and 0.41 (2). The crystal structure features weak C—H⋯O and N—H⋯N inter­actions involving the carbonyl and cyano groups as acceptors.

## Related literature

For biological properties of *N*-pyrazole derivatives, see: Cheng *et al.* (2008 ▶); Liu *et al.* (2010 ▶); Hatton *et al.* (1993 ▶). For related structures, see: Yang *et al.* (2004 ▶); Zhang *et al.* (2005 ▶); Zhong *et al.* (2004 ▶).
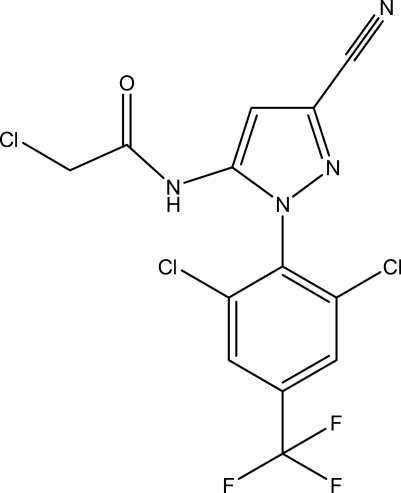


## Experimental

### 

#### Crystal data

C_13_H_6_Cl_3_F_3_N_4_O
*M* *_r_* = 397.57
Triclinic, 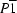

*a* = 8.4190 (17) Å
*b* = 9.2650 (19) Å
*c* = 11.944 (2) Å
α = 69.77 (3)°
β = 76.74 (3)°
γ = 66.10 (3)°
*V* = 794.9 (3) Å^3^
*Z* = 2
Mo *K*α radiation
μ = 0.62 mm^−1^
*T* = 293 K
0.30 × 0.20 × 0.20 mm

#### Data collection

Enraf–Nonius CAD-4 diffractometer
Absorption correction: ψ scan (North *et al.*, 1968 ▶) *T*_min_ = 0.837, *T*_max_ = 0.887
3133 measured reflections
2921 independent reflections
2313 reflections with *I* > 2σ(*I*)
*R*_int_ = 0.030
3 standard reflections every 200 reflections  intensity decay: 1%

#### Refinement

*R*[*F* ^2^ > 2σ(*F* ^2^)] = 0.053
*wR*(*F* ^2^) = 0.155
*S* = 1.01
2921 reflections
246 parameters
H-atom parameters constrained
Δρ_max_ = 0.60 e Å^−3^
Δρ_min_ = −0.58 e Å^−3^

### 

Data collection: *CAD-4 Software* (Enraf–Nonius, 1989 ▶); cell refinement: *CAD-4 Software*; data reduction: *XCAD4* (Harms & Wocadlo, 1995 ▶); program(s) used to solve structure: *SHELXS97* (Sheldrick, 2008 ▶); program(s) used to refine structure: *SHELXL97* (Sheldrick, 2008 ▶); molecular graphics: *SHELXTL* (Sheldrick, 2008 ▶); software used to prepare material for publication: *SHELXL97*.

## Supplementary Material

Crystal structure: contains datablock(s) global, I. DOI: 10.1107/S1600536811052743/bh2393sup1.cif

Structure factors: contains datablock(s) I. DOI: 10.1107/S1600536811052743/bh2393Isup2.hkl

Supplementary material file. DOI: 10.1107/S1600536811052743/bh2393Isup3.cml

Additional supplementary materials:  crystallographic information; 3D view; checkCIF report

## Figures and Tables

**Table 1:** Hydrogen-bond geometry (Å, °)

*D*—H⋯*A*	*D*—H	H⋯*A*	*D*⋯*A*	*D*—H⋯*A*
N4—H4*A*⋯N3^i^	0.86	2.49	3.280 (5)	153
C4—H4*B*⋯O^ii^	0.93	2.53	3.349 (5)	148

Symmetry codes: (i) 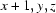; (ii) 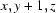.

**Fig. 1.**
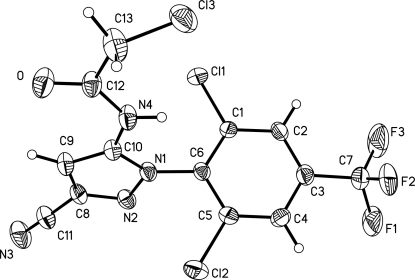
A view of the molecular structure of the title compound. Displacement ellipsoids are drawn at the 50% probability level.

**Fig. 2.**
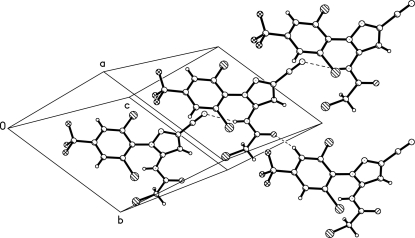
Partial packing view showing the hydrogen-bonded network. Dashed lines indicate intermolecular N—H···N and C—H···O hydrogen bonds.

## supplementary crystallographic information

### Comment

In a variety of biological heterocyclic compounds, *N*-pyrazole derivatives are of great interest because of their chemical and pharmaceutical properties (Cheng *et al.*, 2008). Some X-ray structures of *N*-pyrazole compounds have already been reported (Zhang *et al.*, 2005; Zhong *et al.*, 2004; Yang *et al.*, 2004), and they have been found to exhibit good insecticidal activities against diamond-back moth, mustard beetle, vetch aphid and so on (Hatton *et al.*, 1993). Besides, some other *N*-pyrazole derivatives are known to have antifungal activities (Liu *et al.*, 2010). Herein we report the crystal structure of a new derivative (Fig. 1). In this structure, the pyrazole ring N1/N2/C8/C9/C10 is a planar five-membered ring and the mean deviation from plane is 0.0063 Å. The dihedral angle between the pyrazole and benzene rings is 71.5 (3)°. In the crystal structure, weak intermolecular C—H···O and N—H···N hydrogen bonds (Table 1) link symmetry-related molecules, to form a trimeric unit (Fig. 2), which may be effective in the stabilization of the crystal.

### Experimental

To a stirred solution of 5-amino-1-[2,6-dichloro-4-(trifluoromethyl)phenyl]-1*H*-pyrazole-3-carbonitrile (5 mmol) in THF (20 ml) was added 2-chloroacetyl chloride (5 mmol) dropwise at 0–5 °C. After the addition, the reaction mixture was allowed to rise to room temperature and was stirred for 2 h. The crude product precipitated and was filtered. Pure compound was obtained by crystallization from ethanol. Crystals suitable for X-ray diffraction were obtained by slow evaporation of an acetone solution.

### Refinement

All H atoms bonded to the C atoms were placed geometrically at the distances of 0.93–0.97 Å and included in the refinement in riding motion approximation with *U*_iso_(H) = 1.2 or 1.5*U*_eq_ of the carrier atom. F atoms were disordered over two sites, occupancies were refined and converged to 0.565 (12) and 0.435 (12), respectively.

### Crystal data

**Table:**

C_13_H_6_Cl_3_F_3_N_4_O	*Z* = 2
*M**_r_* = 397.57	*F*(000) = 396
Triclinic, *P*1	*D*_x_ = 1.661 Mg m^−^^3^
Hall symbol: -P 1	Melting point: 483 K
*a* = 8.4190 (17) Å	Mo *K*α radiation, λ = 0.71073 Å
*b* = 9.2650 (19) Å	Cell parameters from 25 reflections
*c* = 11.944 (2) Å	θ = 9–13°
α = 69.77 (3)°	µ = 0.62 mm^−^^1^
β = 76.74 (3)°	*T* = 293 K
γ = 66.10 (3)°	Block, colourless
*V* = 794.9 (3) Å^3^	0.30 × 0.20 × 0.20 mm

### Data collection

**Table:**

Enraf–Nonius CAD-4 diffractometer	2313 reflections with *I* > 2σ(*I*)
Radiation source: fine-focus sealed tube	*R*_int_ = 0.030
graphite	θ_max_ = 25.4°, θ_min_ = 1.8°
ω/2θ scans	*h* = 0→10
Absorption correction: ψ scan (North *et al.*, 1968)	*k* = −10→11
*T*_min_ = 0.837, *T*_max_ = 0.887	*l* = −14→14
3133 measured reflections	3 standard reflections every 200 reflections
2921 independent reflections	intensity decay: 1%

### Refinement

**Table:**

Refinement on *F*^2^	Secondary atom site location: difference Fourier map
Least-squares matrix: full	Hydrogen site location: inferred from neighbouring sites
*R*[*F*^2^ > 2σ(*F*^2^)] = 0.053	H-atom parameters constrained
*wR*(*F*^2^) = 0.155	*w* = 1/[σ^2^(*F*_o_^2^) + (0.1*P*)^2^ + 0.320*P*] where *P* = (*F*_o_^2^ + 2*F*_c_^2^)/3
*S* = 1.01	(Δ/σ)_max_ < 0.001
2921 reflections	Δρ_max_ = 0.60 e Å^−^^3^
246 parameters	Δρ_min_ = −0.58 e Å^−^^3^
0 restraints	Extinction correction: *SHELXL97* (Sheldrick, 2008), Fc^*^=kFc[1+0.001xFc^2^λ^3^/sin(2θ)]^-1/4^
Primary atom site location: structure-invariant direct methods	Extinction coefficient: 0.195 (13)

### Fractional atomic coordinates and isotropic or equivalent isotropic displacement parameters (Å^2^)

**Table:**

	*x*	*y*	*z*	*U*_iso_*/*U*_eq_	Occ. (<1)
O	0.3070 (3)	0.0918 (3)	0.0994 (3)	0.0713 (8)	
Cl1	0.20070 (11)	0.26994 (9)	0.46773 (7)	0.0529 (3)	
Cl2	0.04187 (13)	0.81751 (11)	0.09593 (7)	0.0636 (3)	
Cl3	0.66610 (14)	0.09291 (16)	0.24189 (12)	0.0861 (4)	
N1	0.0425 (3)	0.4846 (3)	0.2382 (2)	0.0414 (6)	
C1	0.1882 (4)	0.4703 (3)	0.3992 (3)	0.0380 (6)	
N2	−0.1343 (3)	0.5475 (3)	0.2373 (2)	0.0462 (6)	
C2	0.2458 (4)	0.5460 (4)	0.4546 (3)	0.0444 (7)	
H2B	0.2910	0.4894	0.5282	0.053*	
C3	0.2352 (4)	0.7066 (4)	0.3993 (3)	0.0480 (7)	
N3	−0.4748 (4)	0.5210 (5)	0.1439 (4)	0.0816 (11)	
N4	0.3004 (3)	0.2913 (4)	0.1692 (3)	0.0510 (7)	
H4A	0.3625	0.3317	0.1884	0.061*	
C4	0.1707 (4)	0.7917 (4)	0.2891 (3)	0.0482 (7)	
H4B	0.1657	0.8996	0.2520	0.058*	
C5	0.1144 (4)	0.7150 (4)	0.2353 (3)	0.0431 (7)	
C6	0.1175 (3)	0.5552 (3)	0.2906 (2)	0.0371 (6)	
C7	0.2921 (6)	0.7937 (5)	0.4592 (5)	0.0759 (12)	
C8	−0.1616 (4)	0.4631 (4)	0.1792 (3)	0.0468 (7)	
C9	−0.0098 (4)	0.3489 (4)	0.1413 (3)	0.0504 (8)	
H9A	0.0011	0.2764	0.0998	0.060*	
C10	0.1202 (4)	0.3680 (4)	0.1793 (3)	0.0419 (7)	
C11	−0.3373 (5)	0.4970 (5)	0.1602 (3)	0.0597 (9)	
C12	0.3826 (4)	0.1554 (4)	0.1304 (3)	0.0511 (8)	
C13	0.5789 (5)	0.0841 (6)	0.1261 (4)	0.0791 (13)	
H13A	0.6268	0.1419	0.0503	0.095*	
H13B	0.6178	−0.0302	0.1271	0.095*	
F1	0.193 (2)	0.9401 (14)	0.447 (3)	0.155 (10)	0.59 (2)
F2	0.4449 (12)	0.8100 (17)	0.3936 (8)	0.104 (3)	0.59 (2)
F3	0.3470 (18)	0.7085 (13)	0.5612 (6)	0.111 (4)	0.59 (2)
F1'	0.1496 (12)	0.851 (3)	0.5471 (17)	0.111 (6)	0.41 (2)
F2'	0.327 (6)	0.913 (4)	0.4027 (11)	0.169 (13)	0.41 (2)
F3'	0.3932 (17)	0.6929 (17)	0.5431 (17)	0.135 (8)	0.41 (2)

### Atomic displacement parameters (Å^2^)

**Table:**

	*U*^11^	*U*^22^	*U*^33^	*U*^12^	*U*^13^	*U*^23^
O	0.0581 (15)	0.0791 (18)	0.097 (2)	−0.0240 (13)	−0.0006 (14)	−0.0539 (16)
Cl1	0.0595 (5)	0.0366 (4)	0.0579 (5)	−0.0150 (3)	−0.0143 (4)	−0.0052 (3)
Cl2	0.0760 (6)	0.0575 (5)	0.0474 (5)	−0.0184 (4)	−0.0215 (4)	0.0000 (4)
Cl3	0.0623 (6)	0.0942 (8)	0.1011 (8)	−0.0006 (5)	−0.0307 (6)	−0.0455 (7)
N1	0.0354 (13)	0.0464 (14)	0.0460 (13)	−0.0133 (11)	−0.0082 (10)	−0.0167 (11)
C1	0.0340 (14)	0.0352 (14)	0.0432 (15)	−0.0093 (11)	−0.0075 (11)	−0.0105 (12)
N2	0.0336 (13)	0.0546 (15)	0.0508 (15)	−0.0133 (11)	−0.0082 (11)	−0.0159 (12)
C2	0.0419 (16)	0.0485 (17)	0.0454 (16)	−0.0113 (13)	−0.0148 (13)	−0.0157 (13)
C3	0.0396 (16)	0.0499 (18)	0.0607 (19)	−0.0134 (14)	−0.0091 (14)	−0.0236 (15)
N3	0.0492 (19)	0.114 (3)	0.097 (3)	−0.0287 (19)	−0.0217 (17)	−0.040 (2)
N4	0.0399 (14)	0.0645 (17)	0.0633 (17)	−0.0206 (12)	−0.0029 (12)	−0.0346 (14)
C4	0.0478 (18)	0.0361 (15)	0.0593 (19)	−0.0151 (13)	−0.0093 (14)	−0.0093 (13)
C5	0.0390 (15)	0.0418 (16)	0.0433 (15)	−0.0090 (12)	−0.0095 (12)	−0.0089 (12)
C6	0.0320 (14)	0.0402 (15)	0.0407 (15)	−0.0103 (11)	−0.0059 (11)	−0.0150 (12)
C7	0.075 (3)	0.058 (2)	0.112 (4)	−0.018 (2)	−0.040 (3)	−0.031 (2)
C8	0.0410 (17)	0.0599 (19)	0.0465 (16)	−0.0212 (14)	−0.0118 (13)	−0.0149 (14)
C9	0.0486 (18)	0.063 (2)	0.0535 (18)	−0.0246 (15)	−0.0079 (14)	−0.0256 (15)
C10	0.0407 (16)	0.0487 (16)	0.0420 (15)	−0.0174 (13)	−0.0047 (12)	−0.0174 (13)
C11	0.053 (2)	0.077 (2)	0.058 (2)	−0.0258 (18)	−0.0128 (16)	−0.0231 (18)
C12	0.0449 (18)	0.063 (2)	0.0556 (19)	−0.0200 (16)	0.0018 (14)	−0.0334 (16)
C13	0.044 (2)	0.116 (4)	0.095 (3)	−0.013 (2)	−0.0016 (19)	−0.072 (3)
F1	0.120 (7)	0.087 (7)	0.31 (3)	0.014 (6)	−0.107 (12)	−0.124 (12)
F2	0.106 (6)	0.127 (7)	0.122 (5)	−0.082 (5)	−0.039 (4)	−0.020 (5)
F3	0.200 (10)	0.138 (7)	0.059 (4)	−0.118 (8)	−0.023 (4)	−0.027 (4)
F1'	0.075 (5)	0.149 (12)	0.155 (11)	−0.020 (6)	−0.009 (5)	−0.124 (10)
F2'	0.35 (4)	0.16 (2)	0.100 (7)	−0.21 (3)	−0.044 (17)	−0.007 (11)
F3'	0.074 (5)	0.128 (9)	0.230 (19)	0.043 (7)	−0.106 (8)	−0.119 (12)

### Geometric parameters (Å, °)

**Table:**

O—C12	1.205 (4)	N4—H4A	0.8600
Cl1—C1	1.720 (3)	C4—C5	1.368 (4)
Cl2—C5	1.717 (3)	C4—H4B	0.9300
Cl3—C13	1.747 (4)	C5—C6	1.390 (4)
N1—C10	1.353 (4)	C7—F2'	1.192 (11)
N1—N2	1.362 (3)	C7—F1	1.249 (8)
N1—C6	1.420 (4)	C7—F3	1.273 (9)
C1—C2	1.382 (4)	C7—F3'	1.304 (13)
C1—C6	1.387 (4)	C7—F2	1.381 (9)
N2—C8	1.319 (4)	C7—F1'	1.454 (11)
C2—C3	1.378 (5)	C8—C9	1.389 (5)
C2—H2B	0.9300	C8—C11	1.437 (4)
C3—C4	1.383 (5)	C9—C10	1.369 (4)
C3—C7	1.504 (5)	C9—H9A	0.9300
N3—C11	1.137 (5)	C12—C13	1.506 (5)
N4—C12	1.353 (4)	C13—H13A	0.9700
N4—C10	1.387 (4)	C13—H13B	0.9700
			
C10—N1—N2	111.9 (2)	F2'—C7—F1'	103.7 (13)
C10—N1—C6	130.1 (2)	F3'—C7—F1'	91.6 (9)
N2—N1—C6	117.8 (2)	F2'—C7—F3'	114.9 (16)
C2—C1—C6	120.8 (3)	F1—C7—C3	112.7 (5)
C2—C1—Cl1	119.3 (2)	F2—C7—C3	106.0 (6)
C6—C1—Cl1	119.9 (2)	F3—C7—C3	115.0 (5)
C8—N2—N1	103.3 (2)	F1'—C7—C3	107.6 (5)
C3—C2—C1	119.0 (3)	F2'—C7—C3	120.9 (7)
C3—C2—H2B	120.5	F3'—C7—C3	113.0 (7)
C1—C2—H2B	120.5	N2—C8—C9	113.8 (3)
C2—C3—C4	121.2 (3)	N2—C8—C11	119.2 (3)
C2—C3—C7	120.2 (3)	C9—C8—C11	127.1 (3)
C4—C3—C7	118.6 (3)	C10—C9—C8	103.8 (3)
C12—N4—C10	122.7 (3)	C10—C9—H9A	128.1
C12—N4—H4A	118.6	C8—C9—H9A	128.1
C10—N4—H4A	118.6	N1—C10—C9	107.2 (3)
C5—C4—C3	119.0 (3)	N1—C10—N4	120.4 (3)
C5—C4—H4B	120.5	C9—C10—N4	132.4 (3)
C3—C4—H4B	120.5	N3—C11—C8	178.4 (4)
C4—C5—C6	121.2 (3)	O—C12—N4	123.4 (3)
C4—C5—Cl2	119.2 (2)	O—C12—C13	120.0 (3)
C6—C5—Cl2	119.6 (2)	N4—C12—C13	116.7 (3)
C1—C6—C5	118.7 (3)	C12—C13—Cl3	115.8 (2)
C1—C6—N1	121.4 (3)	C12—C13—H13A	108.3
C5—C6—N1	119.8 (3)	Cl3—C13—H13A	108.3
F1—C7—F2	102.2 (9)	C12—C13—H13B	108.3
F1—C7—F3	118.1 (10)	Cl3—C13—H13B	108.3
F3—C7—F2	100.1 (6)	H13A—C13—H13B	107.4
			
C10—N1—N2—C8	1.6 (3)	C4—C3—C7—F1	39.4 (16)
C6—N1—N2—C8	177.3 (3)	C2—C3—C7—F3	−0.4 (9)
C6—C1—C2—C3	−1.3 (4)	C4—C3—C7—F3	178.8 (8)
Cl1—C1—C2—C3	−179.9 (2)	C2—C3—C7—F3'	18.7 (11)
C1—C2—C3—C4	−1.0 (5)	C4—C3—C7—F3'	−162.0 (10)
C1—C2—C3—C7	178.2 (3)	C2—C3—C7—F2	109.2 (6)
C2—C3—C4—C5	0.9 (5)	C4—C3—C7—F2	−71.6 (6)
C7—C3—C4—C5	−178.3 (3)	C2—C3—C7—F1'	−80.8 (11)
C3—C4—C5—C6	1.4 (5)	C4—C3—C7—F1'	98.4 (11)
C3—C4—C5—Cl2	−177.6 (2)	N1—N2—C8—C9	−0.6 (4)
C2—C1—C6—C5	3.5 (4)	N1—N2—C8—C11	−179.8 (3)
Cl1—C1—C6—C5	−177.8 (2)	N2—C8—C9—C10	−0.6 (4)
C2—C1—C6—N1	−173.9 (2)	C11—C8—C9—C10	178.6 (3)
Cl1—C1—C6—N1	4.8 (4)	N2—N1—C10—C9	−2.0 (3)
C4—C5—C6—C1	−3.6 (4)	C6—N1—C10—C9	−177.0 (3)
Cl2—C5—C6—C1	175.4 (2)	N2—N1—C10—N4	179.1 (3)
C4—C5—C6—N1	173.8 (3)	C6—N1—C10—N4	4.0 (5)
Cl2—C5—C6—N1	−7.1 (4)	C8—C9—C10—N1	1.5 (4)
C10—N1—C6—C1	−76.2 (4)	C8—C9—C10—N4	−179.8 (3)
N2—N1—C6—C1	109.0 (3)	C12—N4—C10—N1	168.1 (3)
C10—N1—C6—C5	106.4 (4)	C12—N4—C10—C9	−10.5 (6)
N2—N1—C6—C5	−68.3 (3)	C10—N4—C12—O	1.4 (6)
C2—C3—C7—F2'	161 (3)	C10—N4—C12—C13	−179.0 (3)
C4—C3—C7—F2'	−20 (3)	O—C12—C13—Cl3	−143.6 (4)
C2—C3—C7—F1	−139.8 (16)	N4—C12—C13—Cl3	36.7 (5)

### Hydrogen-bond geometry (Å, °)

**Table:**

*D*—H···*A*	*D*—H	H···*A*	*D*···*A*	*D*—H···*A*
N4—H4A···N3^i^	0.86	2.49	3.280 (5)	153.
C4—H4B···O^ii^	0.93	2.53	3.349 (5)	148.

Symmetry codes: (i) *x*+1, *y*, *z*; (ii) *x*, *y*+1, *z*.
